# Brain-age in midlife is associated with accelerated biological aging and cognitive decline in a longitudinal birth cohort

**DOI:** 10.1038/s41380-019-0626-7

**Published:** 2019-12-10

**Authors:** Maxwell L. Elliott, Daniel W. Belsky, Annchen R. Knodt, David Ireland, Tracy R. Melzer, Richie Poulton, Sandhya Ramrakha, Avshalom Caspi, Terrie E. Moffitt, Ahmad R. Hariri

**Affiliations:** 1 Department of Psychology & Neuroscience, Duke University, Box 104410, Durham, NC 27708 USA; 2 Department of Epidemiology, Columbia University Mailman School of Public Health, New York, NY USA; 3 Robert N. Butler Columbia Aging Center, Columbia University, New York, NY USA; 4 Dunedin Multidisciplinary Health and Development Research Unit, Department of Psychology, University of Otago, 163 Union St E, Dunedin, 9016 New Zealand; 5 New Zealand Brain Research Institute, Christchurch, New Zealand; 6 Department of Medicine, University of Otago, Christchurch, New Zealand; 7 Social, Genetic & Developmental Psychiatry Research Centre, Institute of Psychiatry, Psychology & Neuroscience, King’s College London, De Crespigny Park, Denmark Hill, London, SE5 8AF UK; 8 Department of Psychiatry & Behavioral Sciences, Duke University School of Medicine, Durham, NC 27708 USA; 9 Center for Genomic and Computational Biology, Duke University, Box 90338, Durham, NC 27708 USA

**Keywords:** Predictive markers, Neuroscience

## Abstract

An individual’s brainAGE is the difference between chronological age and age predicted from machine-learning models of brain-imaging data. BrainAGE has been proposed as a biomarker of age-related deterioration of the brain. Having an older brainAGE has been linked to Alzheimer’s, dementia, and mortality. However, these findings are largely based on cross-sectional associations which can confuse age differences with cohort differences. To illuminate the validity of brainAGE as a biomarker of accelerated brain aging, a study is needed of a large cohort all born in the same year who nevertheless vary on brainAGE. In the Dunedin Study, a population-representative 1972–73 birth cohort, we measured brainAGE at age 45 years, as well as the pace of biological aging and cognitive decline in longitudinal data from childhood to midlife (*N* = 869). In this cohort, all chronological age 45 years, brainAGE was measured reliably (ICC = 0.81) and ranged from 24 to 72 years. Those with older midlife brainAGEs tended to have poorer cognitive function in both adulthood and childhood, as well as impaired brain health at age 3. Furthermore, those with older brainAGEs had an accelerated pace of biological aging, older facial appearance, and early signs of cognitive decline from childhood to midlife. These findings help to validate brainAGE as a potential surrogate biomarker for midlife intervention studies that seek to measure dementia-prevention efforts in midlife. However, the findings also caution against the assumption that brainAGE scores represent only age-related deterioration of the brain as they may also index central nervous system variation present since childhood.

## Introduction

While old age is associated with higher risk for disease across the entire body, degeneration of the brain and consequent cognitive decline has an outsized influence on disability and loss of independence in older adults [1]. As such there is growing need for interventions to slow the progression of cognitive decline. Unfortunately, to date, tested interventions have not slowed age-related cognitive decline [2]. The failure of these interventions may be related to their targeting of individuals too late in the aging process after neurodegeneration has become inexorable [3, 4]. Alzheimer’s disease and related dementias (ADRD) arise at the end of a chronic pathophysiological process with preclinical stages emerging decades earlier in life [3]. Evaluating interventions to prevent ADRD onset requires the identification of surrogate biomarkers that index subclinical cognitive decline, neurodegeneration, and accelerated aging of the brain by midlife.

While everyone ages chronologically at the same rate, this is not true biologically; some individuals experience accelerated age-related biological degeneration [5, 6]. For decades, researchers have worked to quantify the rate of biological aging and better understand the mechanisms that generate individual differences in the aging process [7]. The resulting measures of accelerated biological aging have been associated with health span, cognitive decline, cancer risk, and all-cause mortality [5, 6, 8]. However, such aging biomarkers have not directly quantified aging in the organ most directly linked to ADRD, namely the brain. To address this gap, a recently developed measure called “brain-age” has been proposed as a biomarker for accelerated aging of the brain [9, 10]. Brain-age is a relatively novel measure derived from neuroimaging, but its interpretation is uncertain.

Brain-age is estimated by training machine-learning algorithms to predict age from structural magnetic resonance imaging (MRI) data collected in large samples of individuals across a broad age range [11]. These machine-learning algorithms “learn” multivariate patterns from MRI data that are useful in explaining variance in chronological age across individuals. The difference between an individual’s predicted age based on MRI data and their chronological age is called the brain age gap estimate (brainAGE) and is usually interpreted as a measure of accelerated aging of the brain. Older brainAGE has been associated with mild cognitive impairment, ADRD, and mortality [11, 12]. Individuals with an older brainAGE are more likely to have risk factors for dementia including obesity, diabetes, alcoholism, and traumatic brain injury [9, 12–14]. Initial studies suggest that brainAGE may be able to predict cognitive decline and conversion to ADRD in older adults in their 60s, 70s, and 80s [15, 16]. But there is no evidence linking brainAGE to earlier signs of cognitive decline or accelerated aging in midlife, the age when surrogate biomarkers may be more effectively used in ADRD-prevention efforts [4]. Promising results notwithstanding, research on brainAGE is still in its infancy. Reported associations between brainAGE and risk factors for accelerated aging are largely cross-sectional. Inferring within-subject decline and aging from cross-sectional associations in people of different-age cohorts has many pitfalls and is prone to confuse aging with cohort differences (e.g., Intelligence Quotient (IQ) scores are higher in members of more recent cohorts, and there are marked generational differences in exposure to diseases, toxins, antibiotics, education, and nutrition which can influence brain measures, including neuroimaging data) [17–19]. Cross-sectional observations that older brainAGE is associated with ADRD and many of its risk factors are consistent with at least two perspectives on brain aging, each of which has distinct implications.

The first perspective is that older brainAGE could be an indicator of accelerated brain aging that has accumulated over an individual’s lifetime and increases susceptibility to ADRD and age-related cognitive decline. This perspective implies that at some point in early development, all individuals have a brainAGE that is very close to zero. BrainAGE scores then diverge with time from chronological age, as genetic, environmental, and lifestyle factors create variation in the rate of brain aging. Here we will refer to this perspective broadly as the “geroscience perspective” [20]. This perspective is based on the geroscience hypothesis which states that aging is the result of deterioration across multiple organ systems and that furthermore this deterioration is the root cause of age-related disease. It is hypothesized that treatments that can slow this decline will therefore reduce the risk for age-related disease. This theoretical interpretation of brainAGE is the dominant interpretive framework found in the brainAGE literature [10, 11, 21].

The second perspective on brain aging is the “early system-integrity” perspective of cognitive/biological aging [22]. According to this perspective, individuals vary in their brain and body health from the beginning of life. Moreover, according to the system-integrity view, the correlation between brain and body health persists across the lifespan so that both brain and body health predict aging outcomes [23–25]. From this perspective, the reason brainAGE predicts ADRD and mortality later in life is because brainAGE is an indicator of compromised lifelong brain health [26, 27]. Instead of reflecting accelerated brain aging and the brain’s accumulated biological degeneration, an older brainAGE at midlife reflects compromised system integrity that has been present since childhood and stable for decades. Importantly these two perspectives are not mutually exclusive and both may help explain the phenomenon of accelerated brain aging.

Here we tested to what extent older brainAGE is associated with accelerated aging and to what extent older brainAGE reflects stable individual differences in system integrity in the Dunedin Study. First, we hypothesized that if individuals with an older brainAGE have brains that are aging faster, they should also have a body that has aged faster, given that, according to the geroscience perspective, aging is the progressive, generalized deterioration, and loss-of-function across multiple organ systems [28, 29]. Second, we hypothesized that if individuals with older brainAGE have undergone accelerated aging they should show signs of cognitive decline [30]. Third, if older midlife brainAGE represents system integrity from early life, we hypothesized that older brainAGE should be correlated with poorer neurocognitive functioning as assessed already in early childhood.

## Methods

See Supplementary Information for expanded “Methods” section.

### Participants

Participants are members of the Dunedin Longitudinal Study, a representative birth cohort (*N* = 1037; 91% of eligible births; 52% male) born between April 1972 and March 1973 in Dunedin, New Zealand (NZ), who were eligible based on residence in the province and who participated in the first assessment at age 3 years [31]. The cohort represented the full range of socioeconomic status in the general population of NZ’s South Island and as adults matches the NZ National Health and Nutrition Survey on key adult health indicators (e.g., body mass index (BMI), smoking, and GP visits) and the NZ Census of citizens of the same age on educational attainment. The cohort is primarily white (93%), which matches the demographics of the South Island. Assessments were carried out at birth and ages 3, 5, 7, 9, 11, 13, 15, 18, 21, 26, 32, 38, and most recently (completed April 2019) 45 years, when 94% (*N* = 938) of the 997 participants still alive took part. Each participant was brought to the research unit for 1.5 days of interviews and examinations. Written informed consent was obtained from participants and study protocols were approved by the NZ Health and Disability Ethics Committee. Brain imaging was carried out at age 45 years for 875 study members (93% of age-45 participants). Data from six study members were excluded due to major incidental findings or previous head injuries (e.g., large tumors or extensive damage to the brain). This resulted in brain-imaging data for our current analyses from 869 study members, who represented the original cohort (attrition analysis in Supplementary; Supplementary Figs. S1 and S2).

### MRI acquisition

Study participants were scanned using a Siemens Skyra 3T scanner (Siemens Healthcare, Erlangen, Germany) equipped with a 64-channel head/neck coil at the Pacific Radiology imaging center in Dunedin, New Zealand. High resolution structural images were obtained using a T1-weighted MP-RAGE sequence with the following parameters: TR = 2400 ms; TE = 1.98 ms; 208 sagittal slices; flip angle, 9°; FOV, 224 mm; matrix = 256 × 256; slice thickness = 0.9 mm with no gap (voxel size 0.9 × 0.875 × 0.875 mm); and total scan time = 6 min and 52 s.

### BrainAGE

We generated brainAGE scores using a recently published, publicly available algorithm [13]. This method uses a stacked algorithm to predict chronological age from multiple measures of brain structure derived from Freesurfer version 5.3 [32]. Specifically, the algorithm is trained on vertex-wise cortical thickness and surface area data extracted from fsaverage4 standard space as well as subcortical volume extracted from the aseg parcellation. Test–retest reliability was assessed in 20 Dunedin Study members (mean interval between scans = 79 days). The ICC of brainAGE was 0.81 (95% CI = 0.59–0.92; *p* < 0.001), indicating excellent reliability [33]. Moreover, we chose this algorithm because of its performance in predicting chronological age in independent samples and its sensitivity to age-related cognitive impairment in old age [13]. All regression analyses used brainAGE scores (i.e., the difference between an individual’s predicted age from MRI data and their exact chronological age, between birth, and the date of the MRI scan).

### Adulthood measures of cognitive functioning and accelerated aging

*Cognitive functioning* at age 45 was assessed with the Wechsler Adult Intelligence Scale-IV [34], which measures the IQ and four specific domains of cognitive function: verbal comprehension, perceptual reasoning, working memory, and processing speed. Study members were also tested with an additional suite of measures of vocabulary, memory, and executive functioning (Table 1 and Supplementary). *Accelerated aging* was assessed (a) by the pace of aging, a longitudinal composite of multiple biomarkers that indexes the integrity of metabolic, cardiovascular, respiratory, kidney, immune, and dental systems, measured at four study waves from the cohort members’ 20s to their mid-40s, and (b) by independent ratings of facial aging. All measures are described in Table 1.

**Table 1:** Description of Study Measures.

Measure	Description
Adult cognitive assessment	
Adulthood IQ	IQ at age 45 was measured with the Wechsler Adult Intelligence Scale-IV (WAIS-IV) [53]. The WAIS-IV generates the overall full-scale IQ, and four WAIS-IV indexes assess abilities that make up the IQ: processing speed, working memory, perceptual reasoning, and verbal comprehension. In addition, we examine performance on the digit symbol substitution [54] subtest, which is most representative of “fluid” cognitive ability, and on the Rey Auditory Verbal Learning test [55] of memory.
Childhood assessments	
Childhood brain health (age 3)	Age-3 Brain Health is a composite measure from a 45-min examination that included assessments by a pediatric neurologist, standardized tests of intelligence, receptive language, and motor skills, and examiners’ ratings of each child’s emotional and behavioral regulation [35].
Childhood IQ	IQ was measured with the Wechsler Intelligence Scale for Children-Revised (WISC-R [36]; averaged across ages 7, 9, and 11). In addition, we examine performance on the digit-span subtest and, the Rey Auditory Verbal Learning test [55] of memory.
Measures of accelerated aging	
Pace of aging	Pace of aging was measured for each Dunedin Study member with repeated assessments of a panel of 19 biomarkers taken at ages 26, 32, 38, and 45 years (see Supplementary for details), as previously described [6].
Facial aging	Facial aging was based on ratings by an independent panel of 8 raters of each Study member’s facial photograph.

### Childhood measures of brain health and cognitive functioning

At age 3 years, each child participated in a 45-min examination that included assessment by a pediatric neurologist and standardized tests of intelligence, receptive language, and motor skills. Afterwards the examiners (having no prior knowledge of the child) rated each child’s emotional and behavioral regulation during the protocol. These five measures were combined to yield an index of age-3 Brain Health (Table 1 and Supplementary) [35]. In late childhood (ages 7, 9, and 11 years), Study members were administered the Wechsler Intelligence Scale for Children-Revised (WISC-R) yielding IQ scores [36]. Scores from the three WISC-R administrations were averaged to yield a single, reliable measure of childhood cognitive function. Study members were also tested with an additional suite of measures of vocabulary, memory, and executive functioning (Table 1).

### Statistical analysis

We tested associations between brainAGE and all target variables using linear regression models in R (version 3.4.0). All models were adjusted for sex. Cognitive decline from childhood to adulthood was measured using a statistical adjustment approach that tested deviation (or change) in a participants’ adult IQ from what would be expected based on their childhood IQ. The premise and analysis plan for this project were preregistered on https://sites.google.com/site/dunedineriskconceptpapers/elliott. Analyses reported here were checked for reproducibility by an independent data analyst, who recreated the code by working from the paper and applied it to an independent copy of the dataset.

## Results

### People of the same chronological age differ in brain-age

**Fig. 1:**
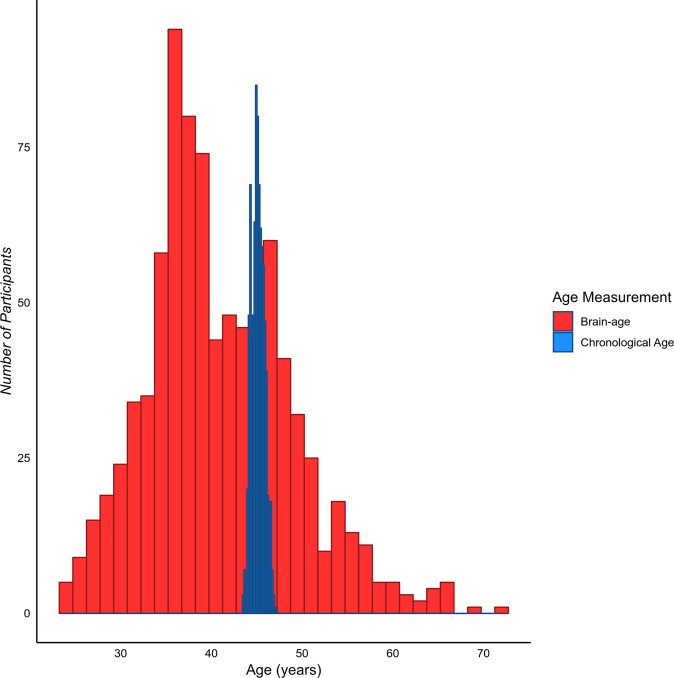
The distribution of chronological age and brain-age amongst the Dunedin Study members. While there is very little variation in chronological age, there is a large amount of variation in brain-age.

As illustrated in Fig. 1, despite the narrow range of chronological ages in the Dunedin Study (mean = 45.15, SD = 0.69, range = 43.48–46.98), there was substantial variation in brain-age (mean = 40.93, SD = 8.04, range = 23.84–71.63). The slight bias towards lower predicted brain-age in this midlife cohort (i.e., we observe younger mean brain-age than mean chronological age) is consistent with findings in this field of research, where brain-age algorithms appear to systematically overestimate mean brain predicted age before age 35 and underestimate mean brain predicted age after age 35 [37].

### Older brainAGE and adult cognitive function

**Fig. 2:**
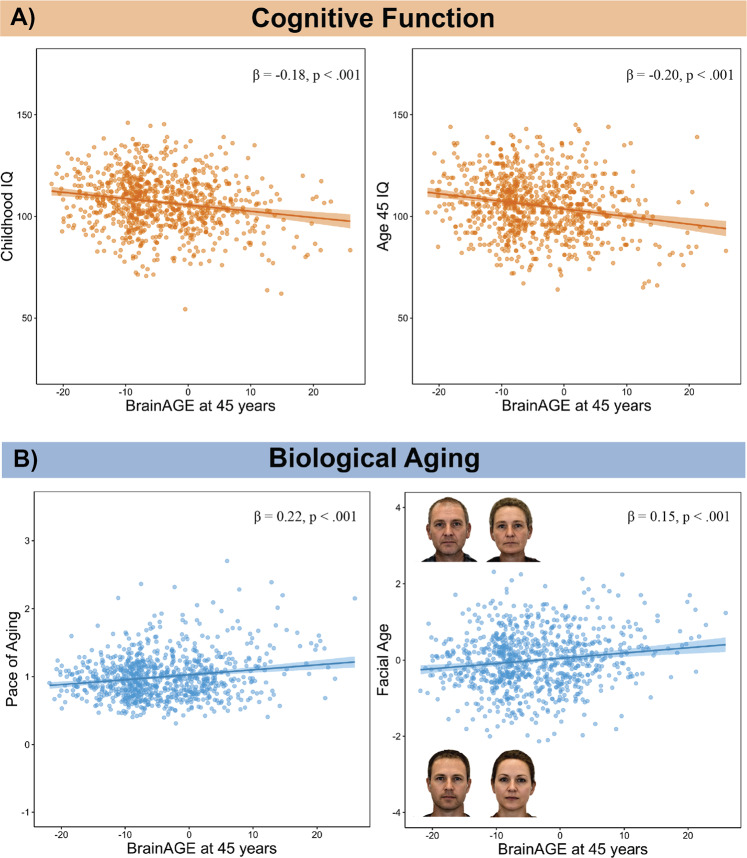
Associations betweeen brainAGE, cognitive function and biological aging. **a** Associations between older age-45 brainAGE and lower cognitive function. The left panel displays the association between older brainAGE and lower childhood IQ. The right panel displays the association between older brainAGE and lower IQ measured at age 45. **b** Associations between older age-45 brainAGE and accelerated biological aging. The left panel displays the association between accelerated pace of biological aging between ages 26 and 45 and older brainAGE. The pace of aging quantifies study members’ rate of biological aging in year‐equivalent units of physiological decline occurring per chronological year. The average study member experienced 1 year of physiological decline per each chronological year, a pace of aging of 1. The right panel displays the association between older facial age and older brainAGE. To illustrate facial aging, the right panel shows digitally averaged faces of the ten male and female Study members rated as looking the oldest and the ten male and female Study members rated as looking the youngest. Facial Age is standardized to *M* = 0, SD = 1.

Both the system-integrity and geroscience perspectives predict that brainAGE should be associated with cognitive function. Consistent with both perspectives, Study members with older brainAGEs performed more poorly on cognitive tests (Table 1). Those with older brainAGE had lower full-scale IQ at age 45 (standardized *β* = −0.20, 95% CI = −0.27 to −0.14; *p* < 0.001; Fig. 2). However, the associations between brainAGE and cognitive functions were nonspecific; Study members with older brainAGEs had lower scores on all IQ subscales at age 45 including verbal comprehension, which is a crystallized measure (standardized *β* = −0.19, 95% CI = −0.26 to −0.13; *p* < 0.001), and the three fluid measures: perceptual reasoning (standardized *β* = −0.17, 95% CI = −0.23 to −0.10; *p* < 0.001), processing speed (standardized *β* = −0.12, 95% CI = −0.19 to −0.05; *p* < 0.001), and working memory (standardized *β* = −0.15, 95% CI = −0.22 to −0.09; *p* < 0.001). In addition, Study members with older brainAGEs performed more poorly on additional cognitive tests, including digit symbol coding (standardized *β* = −0.15, 95% CI = −0.22 to −0.08; *p* < 0.001), as well as tests of memory (Rey total learning: standardized *β* = −0.14, 95% CI = −0.21 to −0.07; *p* < 0.001; and Rey delayed-recall scores: standardized *β* = −0.09, 95% CI = −0.16 to −0.02; *p* = 0.012).

### Older brainAGE, childhood cognitive function, and age-3 Brain Health

The system-integrity perspective predicts that associations between brainAGE and cognitive functions are present since childhood. Consistent with this prediction, 45 years old with older brainAGE had lower full-scale IQ when measured in late childhood (standardized *β* = −0.18, 95% CI = −0.24 to −0.11; *p* < 0.001; Fig. 2). Again we did not find evidence for specificity of this association. Study members with older brainAGE had lower performance IQ, a fluid measure (standardized *β* = −0.14, 95% CI = −0.21 to −0.08; *p* < 0.001), and lower verbal IQ, a crystallized measure (standardized *β* = −0.17, 95% CI = −0.24 to −0.11; *p* < 0.001). As in adulthood, study members with older brainAGE had poorer performance in childhood on digit symbol coding (standardized *β* = −0.09, 95% CI = −0.15 to −0.02; *p* = 0.014). Those with older brainAGE also had poorer performance on measures of memory in childhood (Rey total learning: standardized *β* = −0.13, 95% CI = −0.20 to −0.05; *p* < 0.001; Rey delayed-recall scores: standardized *β* = −0.11, 95% CI = −0.18 to −0.04; *p* < 0.001). Finally, consistent with the system-integrity perspective, Study members with older brainAGEs at age 45 had poorer age-3 Brain Health (standardized *β* = −0.12, 95% CI = −0.19 to −0.05; *p* < 0.001).

### Older brainAGE is associated with accelerated biological aging

The geroscience perspective predicts that Study members with older brainAGEs should have bodies that are aging at a faster rate. We found evidence to support this account as Study members with older brainAGE tended to have a faster pace of aging from age 26 to 45 (standardized *β* = 0.22, 95% CI = 0.15–0.28; *p* < 0.001; Fig. 2). Study members in the oldest decile of brainAGE aged 1.17 biological years per chronological year between ages 26 and 45 years, compared with just 0.95 biological years per chronological year for those in the youngest decile. This amounted to 4.22 additional years of biological aging, between ages 26 and 45, for those in the highest brainAGE decile. Furthermore, those with older brainAGE were rated by independent raters as looking physically older than those with younger brainAGE (standardized *β* = 0.15, 95% CI = 0.09–0.22; *p* < 0.001; Fig. 2). In addition Study members with older brainAGE declined faster in their facial age scores between age 38 and 45 (standardized *β* = 0.07, 95% CI = 0.02–0.12; *p* = 0.009), suggesting older brainAGE predicted a faster pace of facial aging over the course of just 7 years.

### Older brainAGE and accelerated cognitive aging

**Fig. 3:**
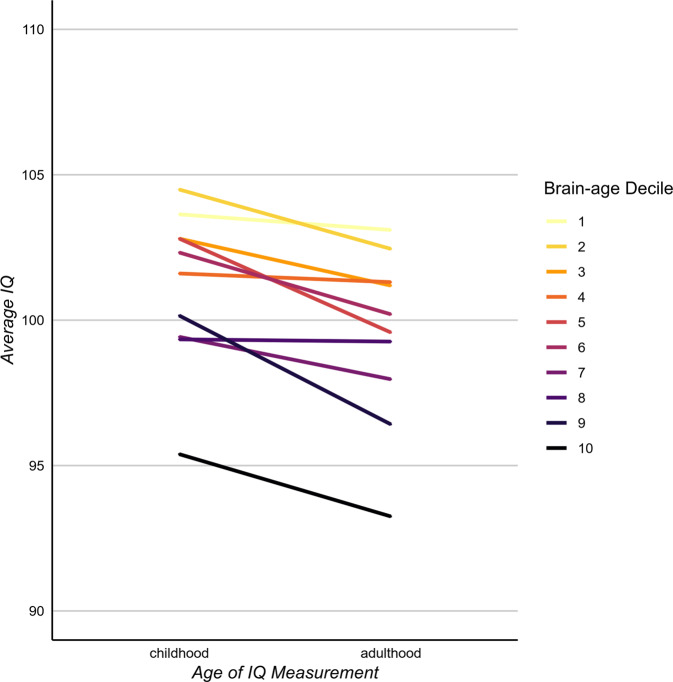
The associations of brainAGE with cognitive functioning and cognitive decline. Those with younger age-45 brainAGE had the highest IQ scores in both childhood and adulthood. In addition, cognitive decline was greatest among those with older age-45 brainAGE; the slopes connecting childhood to adulthood are steeper among Study members with older brainAGEs. Sample sizes for each decile from the lowest to the highest brainAGE were: 86, 86, 85, 86, 85, 86, 86, 85, 86, and 86.

Finally, the geroscience perspective also predicts that Study members with older brainAGE should show cognitive decline. Consistent with this perspective, Study members with older brainAGE showed initial signs of cognitive decline from their childhood IQ scores to their age-45 IQ scores (standardized *β* = −0.07, 95% CI = −0.12 to −0.03; *p* = 0.001; Fig. 3). This decline was also found in cognitive tests known to be especially sensitive to aging-related cognitive decline [38] including digit symbol coding (standardized *β* = −0.10, 95% CI = −0.15 to −0.04; *p* < 0.001) and memory tests (Rey total learning: standardized *β* = −0.12, 95% CI = −0.19 to −0.05; *p* < 0.001; Rey delayed recall: standardized *β* = −0.08, 95% CI = −0.15 to −0.01; *p* = 0.028).

### Sensitivity analyses

Sensitivity analyses (Supplementary Table S1) revealed that brainAGE was associated with adult IQ, cognitive decline and the pace of aging after controlling for brain volume, cerebrospinal fluid volume and intracranial volume. These results suggest that brainAGE measures unique variation in cognition and biological aging over and above commonly used brain measures. Correction for multiple comparisons across all results in Table 2 was done using the false discovery rate. All *p* values remained significant at *p* < 0.05.

**Table 2:** Associations between brainAGE at 45 years, measures of cognitive functioning, accelerated aging, and cognitive decline.

Variable	*n*	Standardized *β* (95% CI)	*P* value
Adulthood cognitive function, age 45			
IQ	867	−0.20 (−0.27 to −0.14)	<0.001
Processing speed	867	−0.12 (−0.19 to −0.05)	<0.001
Working memory	864	−0.15 (−0.22 to −0.09)	<0.001
Perceptual reasoning	867	−0.17 (−0.23 to −0.10)	<0.001
Verbal comprehension	857	−0.19 (−0.26 to −0.13)	<0.001
RAVL memory test (total score)	867	−0.14 (−0.21 to −0.07)	<0.001
RAVL memory test (recall score)	863	−0.09 (−0.16 to −0.02)	0.012
Digit Symbol Coding	867	−0.15 (−0.22 to −0.08)	<0.001
Childhood cognitive function			
IQ	859	−0.18 (−0.24 to −0.11)	<0.001
Performance IQ	847	−0.14 (−0.21 to −0.08)	<0.001
Verbal IQ	847	−0.17 (−0.24 to −0.11)	<0.001
RAVL memory test (total score)^a^	644	−0.13 (−0.20 to −0.05)	<0.001
RAVL memory test (recall score)^a^	643	−0.11 (−0.18 to −0.04)	0.003
Digit symbol coding	847	−0.09 (−0.15 to −0.02)	0.014
Early childhood neurocognitive status			
Age-3 Brain Health	867	−0.12 (−0.19 to −0.05)	<0.001
Biological aging			
Accelerated pace of aging (age 26–45)	868	0.22 (0.15 to 0.28)	<0.001
Facial age (age 45)	868	0.15 (0.09 to 0.22)	<0.001
Accelerated facial aging (age 38–45)	864	0.07 (0.02 to 0.12)	0.009
Cognitive decline (childhood to age 45)			
IQ	857	−0.07 (−0.12 to −0.03)	0.001
RAVL memory test (total score)^a^	644	−0.12 (−0.19 to −0.05)	0.001
RAVL memory test (recall score)^a^	643	−0.08 (−0.15 to −0.01)	0.028
Digit symbol coding	804	−0.10 (−0.15 to −0.04)	<0.001

*RAVL* Rey Auditory Verbal Learning

All *p* values remained significant (*p*  < 0.05) after adjustment for multiple comparisons across all tests using the false discovery rate

^a^Only children attending the research unit were administered the RAVL, resulting in a smaller sample size with data on this neuropsychological test

## Discussion

Using data from a population-representative longitudinal birth cohort followed over four decades, we compared two perspectives of aging (the “geroscience” and “system-integrity” perspectives) that provide disparate explanations for cross-sectional associations between older brainAGE and age-related health outcomes (e.g., ADRD and mortality). We found evidence to support both perspectives. Specifically, while Study members with older brainAGE had lower cognitive ability in adulthood, they also had poorer cognitive functioning in childhood and poorer brain health already at age 3 years. These findings are consistent with the system-integrity account of brainAGE as representing long-standing brain dysfunction present and stable from early life. However, we also found evidence that individual differences in brainAGE were associated with accelerated biological and cognitive aging (e.g., with cognitive decline from childhood to midlife). Together, these findings suggest that an older midlife brainAGE is generated by early individual differences (i.e., system-integrity perspective) as well as by accelerated aging that is accumulated throughout a lifetime (i.e., geroscience perspective).

In addition to comparing perspectives of aging, we were able to investigate the relationship between brainAGE and aging of the rest of the body. By quantifying each person’s personal pace of biological aging, we were able to demonstrate that Study members with older brainAGE had experienced at least two decades of accelerated age-related degradation of the body. Consistent with the “common-cause hypothesis” of aging [28, 39, 40], this finding provides evidence that the brain is not exempt from the biological aging that causes a generalized deterioration of organ systems across the body.

A striking finding in research about aging and mortality is that measures of health taken very early in life can predict the likelihood of death and disease much later in life [23]. For example, individuals with low birthweight are at an increased risk for disease and early mortality [29, 41]. Consistent with these findings we found that brainAGE at age 45 can, in part, be predicted from cognitive function measured in middle childhood and from poor brain health measured at age 3 years. These findings suggest that accelerated brain deterioration and aging, indexed here with brainAGE, may be one mechanism through which individual differences in early system integrity lead to later morbidity and mortality [42, 43]. Further research is needed to test whether brainAGE mediates the relationship between early deficits in system integrity and later age-related disease.

Our study is not without limitations. First, we do not have childhood brain-imaging data that would allow us to directly link accelerated biological aging to accelerated brain aging in the same individuals over time. MRI was not performed in child cohorts during the 1970’s. Previous studies have found that longitudinal changes in brainAGE track changes in symptom severity in schizophrenia and cognitive decline in older adults with ADRD [16, 44] but it is not yet known if changes in brainAGE track with cognitive decline earlier in the life course.

Second, like other studies of brainAGE of which we are aware, the brainAGE metric used here was trained on structural MRI data from a large cross-sectional dataset of individuals across a broad age range [13]. While we have demonstrated that this approach can measure signs of accelerated aging in the brain, it is nevertheless limited in two major ways: (1) brainAGE is based on cross-sectional comparisons of individuals of different ages, which do not distinguish cohort effects (cohort differences in exposures) from developmental changes [17, 18]. As a result brainAGE may be less sensitive to interventions that modify aging processes. (2) BrainAGE incorporates only information from T1-weighted structural scans. Diffusion-weighted imaging, fluid-attenuated inversion recovery, and functional imaging are known to change with advancing age and are linked with aging-related brain disease [45–47]. Integrating these additional data types into brainAGE algorithms may produce biomarkers more predictive of pathogenic brain aging. Optimal brainAGE biomarkers for testing interventions to slow brain aging should be developed from longitudinal, multimodal MRI data that measure accelerated, within-subject brain aging.

While many of the effect sizes observed here are modest, they are based on brainAGE models that do not yet include the rich and more informative data that will become available from longitudinal, multimodal MRI datasets collected across the lifespan [48]. It is not unreasonable to expect incremental improvements in predictive utility and clinical applicability as research on brainAGE expands as has occurred with genome-wide association studies, which have continued to improve the predictive utility of genomic markers with increasing sample sizes especially through data sharing [49].

Prevention of ADRD is a pressing public health priority due to our rapidly aging population and the lack of effective treatments for ADRD in old age [50, 51]. For prevention to be successful, reliable measures are needed of subclinical changes in accelerated brain aging that occur in midlife, decades before the onset of clinically relevant symptoms [3, 52]. Such measures would allow identification of modifiable risk factors, novel treatment targets, and an improved ability to evaluate the effectiveness of preventive interventions. Here we have shown that midlife brainAGE is associated with individual differences in the pace of biological and cognitive aging, suggesting that brainAGE holds promise as a surrogate biomarker for these purposes, and brainAGE measures should continue to be refined. Importantly, we provide evidence that brainAGE is a reliable measure in midlife that demonstrates incremental validity over commonly used brain measures and is indicative of accelerated aging as well as of early system-integrity deficits that may predispose the brain to late-life disease.

## Supplementary information

Supplemental Information
